# The complete chloroplast genome of *Ormosia purpureiflora* (Fabaceae)

**DOI:** 10.1080/23802359.2021.1994901

**Published:** 2021-11-03

**Authors:** Zheng-Feng Wang, Eng-Ping Yu, Qing-Sheng Zeng, Hua-Ge Deng, Hong-Lin Cao, Zhi-An Li, Xiao Wei, Shiou Yih Lee

**Affiliations:** a Guangdong Provincial Key Laboratory of Applied Botany, South China Botanical Garden, Chinese Academy of Sciences, Guangzhou, China; b Center for Plant Ecology, Core Botanical Gardens, Chinese Academy of Sciences, Guangzhou, China; c Southern Marine Science and Engineering Guangdong Laboratory (Guangzhou), Guangzhou, China; d University of Chinese Academy of Sciences, Beijing, China; e Guangdong Forest Resources Conservation Centre, Guangzhou, China; f Guangdong Luofushan Provincial Nature Reserve Management Office, Huizhou, China; g Guangxi Institute of Botany, Chinese Academy of Sciences, Guilin, China; h Faculty of Health and Life Sciences, INTI International University, Nilai, Malaysia

**Keywords:** Chloroplast, endangered species, genome assembly, HiFi sequencing, *Ormosia purpureiflora*

## Abstract

*Ormosia purpureiflora* is endemic to China. It is named after its purple flowers. It is a small tree only up to 3 m. It has leathery leaves, racemose inflorescences. The seeds are elliptic and red in coat. It is only confined to Luofushan Provincial Nature Reserve in Huizhou of Guangdong Province. Herein, we first reported on its complete chloroplast genome sequence as genomic resource for conservation purposes. The chloroplast genome of *O*. *purpureiflora* was 173,364 bp in length, with a large single-copy region of 73,465 bp, a small single-copy region of 18,751 bp, and a pair of inverted repeat regions that were 40,574 bp each. A total of 90 protein-coding genes, 38 transfer RNA genes, and eight ribosomal RNA genes were predicted, while 106 simple sequence repeats were recorded throughout the genome. Phylogenetic analysis revealed that *O*. *purpureiflora* was sister to *O. emarginata*.

*Ormosia* of the family Fabaceae is comprised of about 130 species, which are in forms of trees or shrubs that are widely distributed throughout the tropical regions, with some extended into the temperate zones of East Asia (Liu et al. 2019). The distinct feature for members of *Ormosia* is their red or black seeds that are soughed as raw material for jewelry and ornamental items. Some of the species are classified as important horticulture plants useful for landscaping purposes, afforestation, and source of medicine (Li et al. 2020; Liu et al. 2020). *Ormosia purpureiflora* L. Chen is an endangered species that is endemic to China. It is currently only known to a single location at Luofushan Provincial Nature Reserve in Huizhou of Guangdong Province. It is named after its purple flowers. It is a small tree only up to 3 m. Its leaves are imparipinnate, 5–6 pairs, leathery. It has racemose inflorescences. Its flowers are 5-merous and calyx is campanulate. Its pod is oval or oblong, contains 2–6 seeds. Its seeds are elliptic and red in coat. In order to provide a genomic resource for planning an effective conservation strategy, the complete chloroplast genome of this species was reported in this study.

Leaves of *O*. *purpureiflora* were collected from the Guangdong Luofushan Provincial Nature Reserve (N 23°15′56.90″, E 114°0′25.45″). A voucher specimen was deposited at Herbarium of South China Botanical Garden (http://herbarium.scbg.cas.cn/, Fei-Yan Zeng, zengfeiy@scib.ac.cn) under the voucher number IBSC 0197021. Whole genome sequencing (WGS) was conducted using the PacBio Sequel II system under circular consensus sequencing strategy. As the WGS reads contain both the nuclear and plastid genome sequences, the chloroplast-based reads were extracted by mapping against 10 other *Ormosia* chloroplast genomes available in the NCBI GenBank database. The chloroplast genome sequences were then assembled with extracted reads using Flye 2.8.3 (Kolmogorov et al. 2019). Upon genome assembly, the genes were annotated using both CPGAVAS2 (Shi et al. 2019) and PGA (Qu et al. 2019). The annotated genome was deposited to the NCBI GenBank under the accession number MZ707527. Phylogenetic analysis was performed using Bayesian’s inference method available in PhyloSuite 1.2.2 (Zhang et al. 2020) based on the concatenated sequences of 76 shared chloroplast protein-coding genes in the 11 species of *Ormosia*. Two closely related species, *Lupinus luteus* and *L. westianus* were included as outgroups.

The chloroplast genome of *O*. *purpureiflora* was 173,364 bp in length, with a GC content of 35.81%. The genome contained a large single-copy region of 73,465 bp, a small single-copy region of 18,751 bp, and two copies of inverted repeat regions that each were 40,574 bp in size. A total of 136 genes were predicted, including 90 protein-coding, 38 transfer RNA, and eight ribosomal RNA genes. For the 106 SSRs discovered in the genome, 97 were mononucleotides (A/T), eight were dinucleotides (TA/AT), and one was trinucleotide (ATA). Phylogenetic reconstruction based on the shared protein-coding gene sequences revealed a fully resolved relationship between the 11 species of *Ormosia*, while *O*. *purpureiflora* was sister to *O. emarginata* (Figure 1). The finding of this study could serve as valuable resource for future conservation efforts as well as provide insights to the evolution of *Ormosia* in the family Fabaceae.

**Figure 1.**
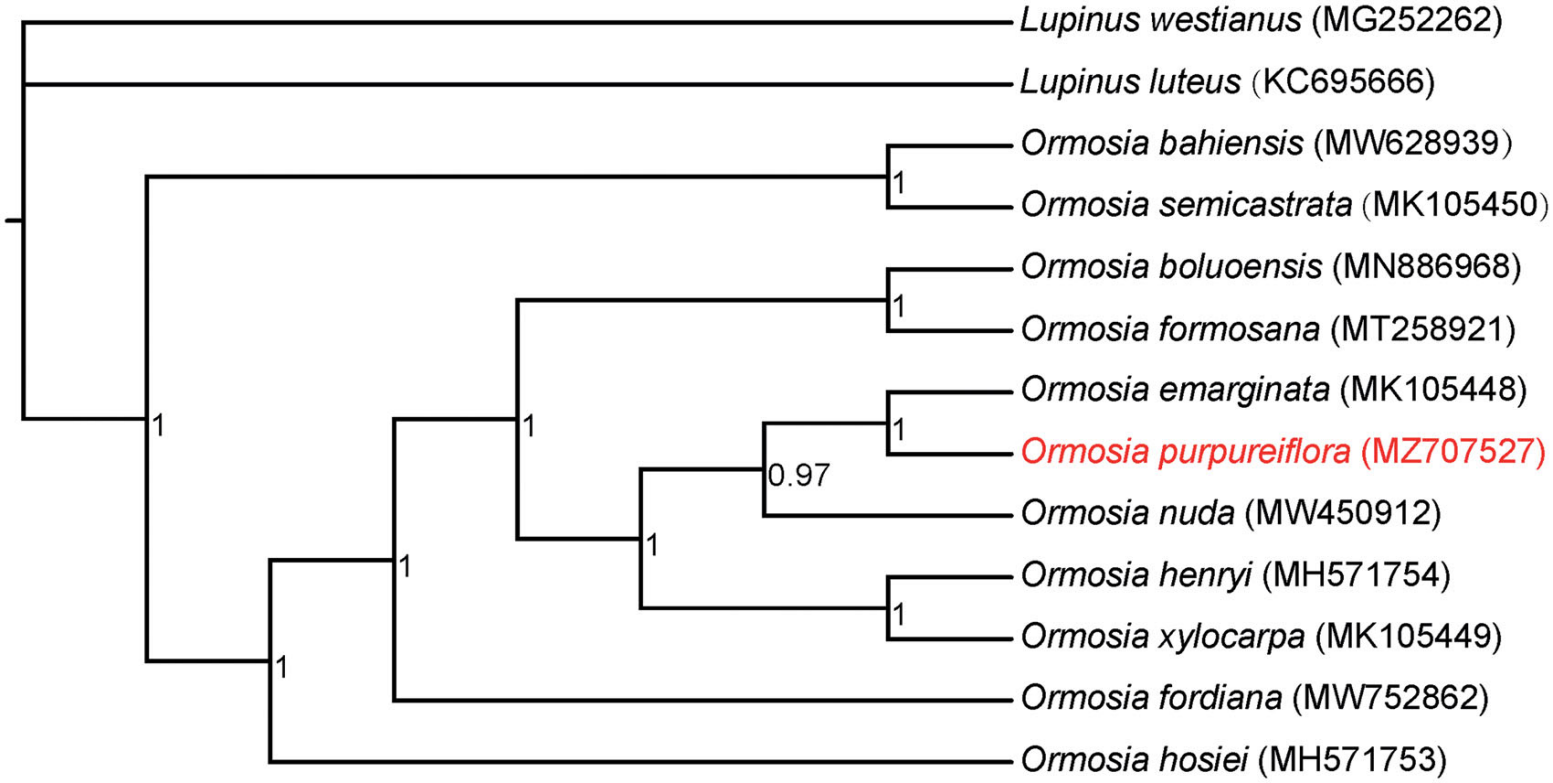
Bayesian’s inference of 11 species of *Ormosia* based on 76 shared protein-coding gene sequences in the chloroplast genome. *Lupinus luteus* and *L. westianus* were included as outgroups. GenBank accession numbers of each species are shown in parentheses. The value of the Bayesian posterior probability is indicated on each branch nodes.

## Data Availability

The genome sequence data that support the finding of this study are openly available in GenBank of NCBI at https://www.ncbi.nlm.nih.gov/ under the accession number MZ707527 and is also accessible at https://doi.org/10.13140/RG.2.2.32309.60643. The associated BioProject, SRA, and Bio-Sample numbers are PRJNA752485, SRR15362743, and SAMN20599437, respectively.
